# First‐line immune checkpoint therapy in metastatic squamous cell lung cancer harboring both *EGFR* mutation and high expression of PD‐L1: A case report

**DOI:** 10.1111/1759-7714.13436

**Published:** 2020-04-14

**Authors:** Kosuke Hamai, Hiroki Tanahashi, Sayaka Ueno, Hanae Konishi, Mirai Matsumura, Akio Nomura, Kanako Nakamoto, Shoko Isoyama, Takuya Tanimoto, Hiroyasu Shoda, Nobuhisa Ishikawa

**Affiliations:** ^1^ Department of Respiratory Medicine Hiroshima Prefectural Hospital Hiroshima Japan; ^2^ Department of Internal and Molecular Medicine Hiroshima University Hiroshima Japan; ^3^ Department of Respiratory Medicine Hiroshima City Asa Citizens Hospital Hiroshima Japan; ^4^ Department of Respiratory Medicine Hiroshima City Funairi Citizens Hospital Hiroshima Japan; ^5^ Department of Respiratory Medicine Hiroshima City Hiroshima Citizens Hospital Hiroshima Japan

**Keywords:** Epidermal growth factor receptor *(EGFR)* mutation, pembrolizumab, programmed death‐ligand 1 (PD‐L1), squamous cell lung cancer

## Abstract

A 90‐year‐old female was admitted to our hospital with a history of a dry cough. Chest computed tomography (CT) scan showed a tumor shadow, and CT‐guided lung biopsy revealed squamous cell carcinoma harboring an *EGFR* mutation. In addition, programmed death‐ligand 1 (PD‐L1) was highly expressed with a tumor proportion score (TPS) of >75%. Pembrolizumab therapy in the first‐line setting was not effective, and the patient died at six months from the first visit. Squamous cell lung cancers (SCLCs) with both *EGFR* mutation and high expression of PD‐L1 are very rare.

## Introduction

Epidermal growth factor receptor (EGFR)‐tyrosine kinase inhibitor (TKI) treatment is effective for lung cancer harboring *EGFR* mutations; a large number of these cases are nonsquamous cell carcinomas. The efficacy of EGFR‐TKIs against squamous cell lung cancer (SCLC) harboring *EGFR* mutations is limited.^1^ Pembrolizumab therapy is recommended in the first‐line setting for lung cancers with high expression of programmed death‐ligand 1 (PD‐L1).^2^ In patients with nonsquamous cell lung cancer harboring *EGFR* mutations and high expression of PD‐L1, EGFR‐TKI therapy is used as the efficacy of pembrolizumab is limited. However, no previous reports have demonstrated the choice of therapy for SCLCs harboring *EGFR* mutations with high expression of PD‐L1.

### Case report

**Figure 1:**
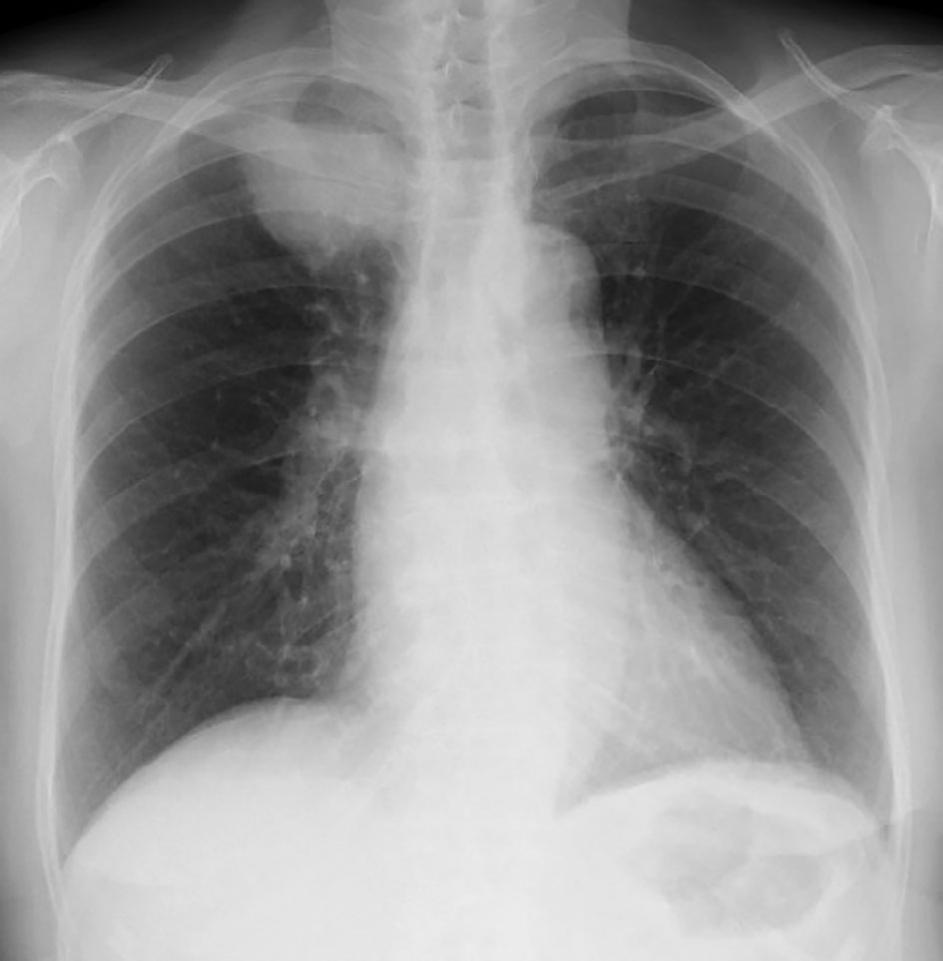
Chest radiograph at hospitalization showed a lung mass in the right upper field.

**Figure 2:**
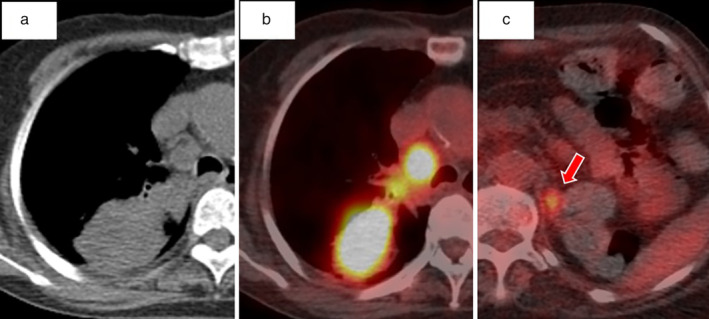
(**a**) Chest unenhanced computed tomography (CT) scan at hospitalization revealed a tumor shadow in the upper lobe of the right lung. Positron emission tomography (PET)‐CT scan before chemotherapy showed SUVmax: (**b**) 26.0 to the mass in the upper lobe of the right lung, and (**c**) 4.8 in the left adrenal gland of with ^18^F‐fluorodeoxyglucose (FDG) integration.

**Figure 3:**
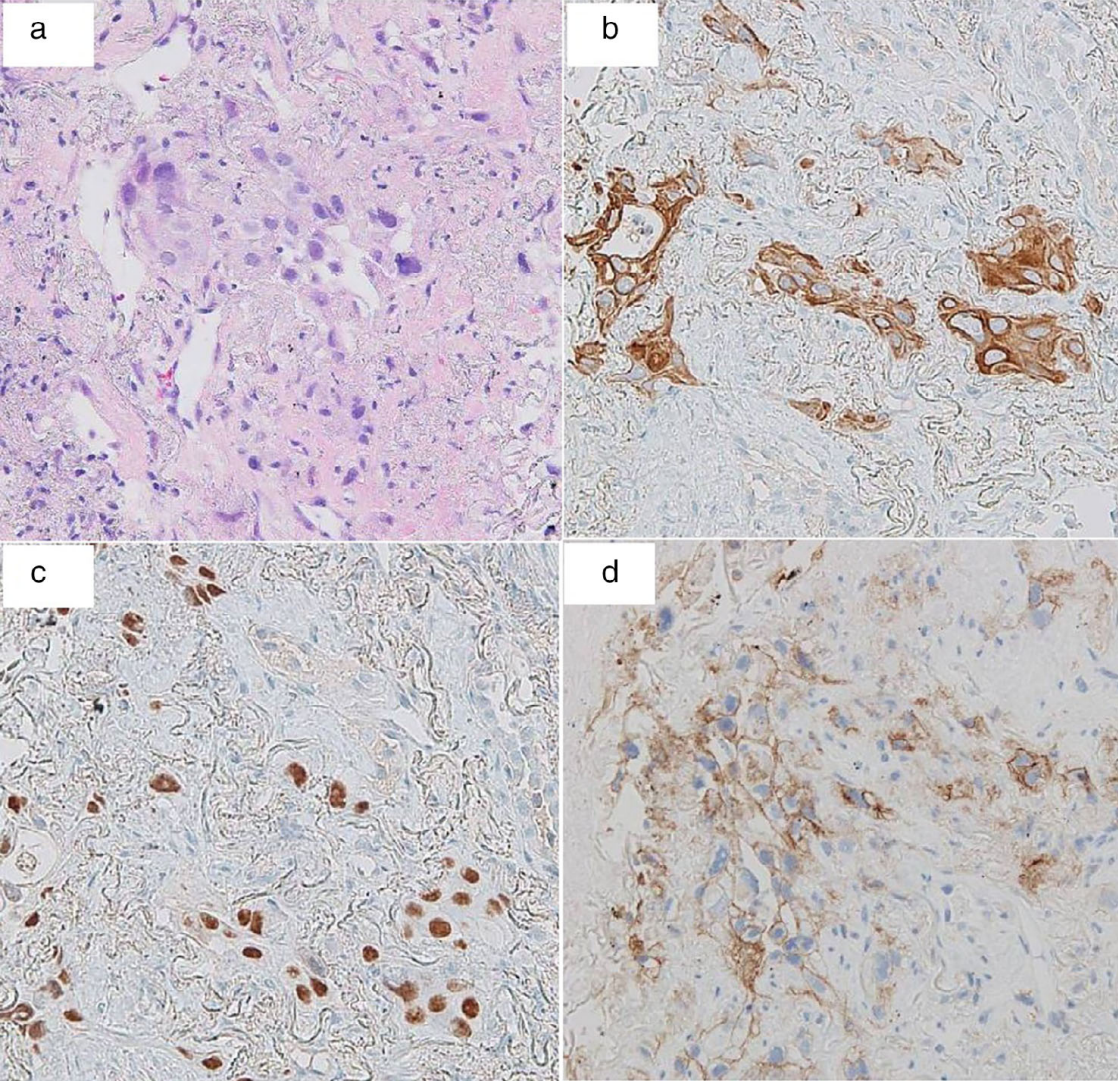
Pathological findings of tumor tissue obtained by CT‐guided needle biopsy showed squamous cell carcinoma. (**a**) Hemotoxylin‐eosin stain revealed that the right lung mass consisted of atypical squamous cells, which was partially positive for (**b**) cytokeratin 5/6 and (**c**) p40. (**d**) Furthermore, programmed death‐ligand 1 (PD‐L1) showed high expression with a tumor proportion score (TPS) >75%.

A 90‐year‐old female was admitted to our hospital with a history of a dry cough. Chest radiograph at hospitalization revealed a lung mass in the right upper field (Fig 1). Chest computed tomography (CT) scan revealed a tumor shadow in the upper lobe of the right lung and swollen mediastinal lymph nodes in the right apical area (Fig 2a). The patient had no history of smoking, and her performance status (PS) score was 1. The serum carcinoembryonic antigen level was 5.5 ng/mL, cytokeratin fragment level was 12.68 ng/mL and progastrin‐releasing peptide level was 83.24 pg/mL. Positron emission tomography (PET)‐CT revealed the maximum standardized ^18^F‐fluorodeoxyglucose uptake value to be 26.0 for the mass in the upper lobe of the right lung, 12.8 for the right hilar lymph nodes, 17.7 for the ipsilateral mediastinal lymph nodes, and 4.8 for the left adrenal gland (Fig 2b,c). Based on the PET‐CT results, cT3N2M1b (ADR), stage IVA lung cancer was suspected. CT‐guided needle biopsy from the tumor in the apical region of the right lung revealed squamous cell carcinoma (Fig 3a–c). The tumor tested positive for *EGFR* mutations (exon 21: L858R) and showed high expression of programmed death‐ligand 1 (PD‐L1), with a tumor proportion score (TPS) of >75% (Fig 3d). Three cycles of pembrolizumab therapy were administered in the first‐line setting. However, the primary lesion, right subclavian and mediastinal lymph node size, and the right‐sided pleural effusion significantly increased. It was difficult to continue treatment owing to poor PS, and the patient died at six months from the first visit.

## Discussion

Epidermal growth factor receptor‐tyrosine kinase inhibitors (EGFR‐TKIs) are effective for nonsmall cell lung cancers harboring *EGFR* mutations, particularly in patients aged >75 years; gefitinib resulted in a progression‐free survival (PFS) of 12.3 months and a 74% objective response rate (ORR) in the study by Goto *et al*.^3^ It has also previously been reported that primary treatment with pembrolizumab for lung cancer with high PD‐L1 expression was better than conventional chemotherapy, with a PFS of 10.3 months, and a six‐month survival rate of 80.2%. In particular, subset analysis of this trial showed pembrolizumab therapy to be more effective for squamous cell carcinoma (SCC) than for nonsquamous cell carcinoma.^2^

The effectiveness of immune checkpoint inhibitors (ICIs) for *EGFR* mutation‐positive lung cancer is limited. In a single‐center retrospective study, the ORR of ICIs for driver mutation‐positive lung cancer was 3.8%.^4^ In contrast, the ORR after using ICIs prior to EGFR‐TKIs was 0%.^5^ Therefore, EGFR‐TKIs are more effective than anti PD‐1 antibodies for nonsquamous cell cancer with both *EGFR* mutations and high expression of PD‐L1.

However, the efficacy of EGFR‐TKI in SCC has been reported to be limited in *EGFR* mutation‐positive cases.^1^ Furthermore, some reports have shown the proportion of *EGFR* mutation‐positive lung cancer with high PD‐L1 expression (≥ 50%) to be approximately 10%; the efficacy of EGFR‐TKIs in such cases were inferior to that observed with lower expression of PD‐L1.^6^, ^7^, ^8^ It was speculated that the efficacy of EGFR‐TKI in our case may be inferior to that mentioned in a previous report on SCLC harboring *EGFR* mutations. Finally, the patient was presented with a choice of injectable treatment or oral medication, and pembrolizumab therapy was selected.

There is no standard treatment for SCLC with *EGFR* mutations and high expression of PD‐L1. In our case, the ICI was administered prior to EGFR‐TKIs owing to its efficacy in SCC, and as per the patient's choice; however, no effect was observed. In this case, the use of cytotoxic anticancer agents was not feasible owing to the age of the patient. The frequency of EGFR‐TKI‐induced interstitial lung disease after ICI use has been reported to increase,^9^ and it was also difficult to administer EGFR‐TKIs when ICI was ineffective. In *EGFR*‐mutated SCLC with high expression of PD‐L1, EGFR‐TKIs as the first‐line treatment followed by ICI may be recommended. Thus, as SCLC with *EGFR* mutations and high expression of PD‐L1 is very rare, further accumulation of treatment experience is required.

## Disclosure

The authors report no conflict of interest.
